# Structural Characterization of Polymer-Clay Nanocomposites Prepared by Co-Precipitation Using EPR Techniques

**DOI:** 10.3390/ma7021384

**Published:** 2014-02-21

**Authors:** Udo Kielmann, Gunnar Jeschke, Inés García-Rubio

**Affiliations:** 1 Laboratory of Physical Chemistry, ETH Zurich, CH-8093 Zurich, Switzerland; E-Mails: udo.kielmann@phys.chem.ethz.ch (U.K); gunnar.jeschke@phys.chem.ethz.ch (G.J.); 2 Centro Universitario de la Defensa, Ctra de Huesca s/n, 50090 Zaragoza, Spain

**Keywords:** EPR, HYSCORE, spin label, polymer, composite, clay, nanocomposite

## Abstract

Polymer-clay nanocomposites (PCNCs) containing either a rubber or an acrylate polymer were prepared by drying or co-precipitating polymer latex and nanolayered clay (synthetic and natural) suspensions. The interface between the polymer and the clay nanoparticles was studied by electron paramagnetic resonance (EPR) techniques by selectively addressing spin probes either to the surfactant layer (labeled stearic acid) or the clay surface (labeled catamine). Continuous-wave (CW) EPR studies of the surfactant dynamics allow to define a transition temperature T* which was tentatively assigned to the order-disorder transition of the surfactant layer. CW EPR studies of PCNC showed that completely exfoliated nanoparticles coexist with agglomerates. HYSCORE spectroscopy in PCNCs showed couplings within the probe −assigned with DFT computations− and couplings with nuclei of the environment, ^1^H and ^23^Na for the surfactant layer probe, and ^29^Si, ^7^Li, ^19^F and ^23^Na for the clay surface probe. Analysis of these couplings indicates that the integrity of the surfactant layer is conserved and that there are sizeable ionic regions containing sodium ions directly beyond the surfactant layer. Simulations of the very weak couplings demonstrated that the HYSCORE spectra are sensitive to the composition of the clay and whether or not clay platelets stack.

## 1. Introduction

The improved characteristics of polymer-clay nanocomposite (PCNC) materials with respect to the pure polymers are more manifest, the better the dispersion of silicate layers into the matrix polymer is [1,2]. Due to the different hydrophobicity of these two components, the two phases are hardly mixed without the participation of surfactants. Based on this idea, one way of producing composites is to use clay that is organically modified by cationic detergents that will act as an interface between the negatively charged silicate layers and the normally apolar polymer. Alternatively, synthetic [3] or natural [4] rubber-clay nanocomposites can be produced by compounding the polymer latex with an aqueous dispersion of pristine clay, which is much less expensive than organically modified clays. Rubber material can be obtained by drying the compounded polymer-clay dispersion [4] or by coagulation [3,5]. In this case, the presence of counterions to compensate for the negative charges of the silicate platelets and, in the case of synthetic rubber, of detergents in the latex is required. These counterions will take part in the interactions connecting the two components, which are, in the end, intimately associated with the macroscopic properties of the material.

XRD (X-Ray Diffraction) and TEM (Transmission Electron Microscopy), the most common techniques used to study polymer-clay nanocomposites—as useful as they are to provide information about the clay interlayer spacing, morphology and the microstructure—cannot give direct evidence for the molecular structure or for dynamics of the interface. Alternatively, specific studies on the surfactant layer have been carried out by paramagnetic labeling of some of its molecules in combination with Electron Paramagnetic Resonance (EPR), which can characterize the motion of the label on the pico- to nanosecond time ranges and give information about the environment of the electron spin from first neighbors to a length scale of up to 10 nanometers [6–10].

Due to its stability, nitroxide molecules are, by far, the most widely used spin probes. Amphoteric molecules with a polar head and an akyl chain with a nitroxide group at a certain carbon of the chain are addressed to the surfactant layer and have been widely used in EPR studies of surfactant micelles, polymer-clay nanocomposites and even biological membranes [6–14]. The working hypothesis is that the polar group of the probe will align with the surface of polar heads of the detergent layer or lipid. CW EPR can give information about the polarity of the probe environment through the sensitivity of the electron gyromagnetic tensor and the nitrogen hyperfine coupling to polarity. Due to the anisotropic spectrum of nitroxides, CW EPR can also provide information about dynamics of the probe in the nanosecond time scale. Detection of the glas-transition temperature or other thermally activated processes, like melting of crystalline domains of polymers or the order-disorder transition of the surfactant layer, have already been successfully detected with EPR spin probing techniques in nanocomposites [6,7]. Additionally, pulse EPR techniques have broadened the scope of the studies by providing information about weak interactions of the electron spin in the paramagnetic probe with other spins in their environment. Interactions with other spin probes—studied with the technique indistinctively called DEER (Double Electron Electron Resonance) or PELDOR (Pulse Electron Double Resonance)—have yielded information on organo-clay particle stack size in melt-compounded microcomposites [8]. The detection of hyperfine interactions of the electron spin with magnetic nuclei can provide information ranging from bonding geometry to atomic distance distributions. To date, ENDOR (Electron Nuclear Double Resonance) experiments have been successfully applied to measure distances in the range 0.3–1 nm from the spin probe to ^31^P-nuclei on TBP surfactants [8]. ESEEM (Electron Spin Echo Envelope Modulation), another other major EPR technique to detect hyperfine interactions, has been used in polymer science to provide qualitative local elemental analysis [6] based on characteristic nuclear Zeeman frequencies. Quantitative information about the number of coupled nuclear spins and their distance to the electron spin is encoded in the modulation depth of the ESEEM traces. However, to date, in PCNC, such quantitative analysis has only been performed to study the accessibility of deuterated compounds to the spin probe [8,10].

HYSCORE (Hyperfine Sublevel Correlation Spectroscopy) is a two-dimensional ESEEM technique that offers increased resolution and is well established in other application fields [15]. It is particularly suited for systems interacting with a large number of different nuclear spins, some of which may have similar Zeeman frequencies, especially if both large and small couplings can be found.

In this paper, we explore the potential of HYSCORE to study the region of contact between polymer and clay phases in polymer-clay nanocomposites produced by co-precipitation of a dispersion containing polymer micelles enclosed by the anionic surfactant sodium dodecyl sulfate (SDS) and exfoliated clay mineral nanoparticles. Synthetic nitrile-butadiene rubber (NBR) latex as well as a poly (methyl methacrylate)-co-(butyl acrylate) dispersion PMMA-co-Bu were used. The contact region can be large, as the required charge-compensating cations will presumably be located also at the interface. Therefore, we probed the two sides of the interface by using different bifunctional nitroxide spin probes which would confer specific affinity to either the surfactant micelle or the clay surface.

## 2. Theory

In the presence of a magnetic field, the two energy levels of an electron spin, *m*_S_ = 1/2 and *m*_S_ = −1/2, are split due to the interaction of the Zeeman interaction between the magnetic moment of the spin and the magnetic field. In EPR spectroscopy, transitions between these two levels are induced by microwave irradiation. If in the vicinity of the electron there is a magnetic nucleus, it will change the local magnetic field at the electron site and therefore it will also change the energy levels of the electron. We say then that the electron and the nucleus are *coupled*. This interaction between electron and nuclear spin is called hyperfine interaction and has several contributions:

- Isotropic or Fermi contact interaction. This contribution is related to the presence of spin density in the nucleus, which can only arise in s orbitals. It is characterized by the isotropic hyperfine coupling *A*_iso_ and is useful to map the electron spin density in a molecule because isotropic couplings are only detectable in atoms directly bearing some spin density;
- Anisotropic or dipole-dipole interaction. This contribution comes from the magnetic interaction between electron and nuclear magnetic dipoles. It can occur between the electron and nucleus of an atom bearing a nuclear spin if it has some spin density in nonspherical orbitals (p, d, f), or as a result of through-space coupling between an electron and the nuclear spin of a neighboring atom. If the distance between electron spin center and nucleus, *r*, is 2.5 Å or more, the unpaired electron density can be considered to be concentrated at one point and the hyperfine tensor can be approximated by the point-dipole expression:
  $$T=\frac{\mu_{0}}{4\pi h}\frac{g_{\text{e}}\beta_{\text{e}}g_{\text{n}}\beta_{\text{n}}}{r^{3}}\left(\begin{array}{ccc} -1 & & \\ & -1 & \\ & & 2 \end{array}\right)=\left(\begin{array}{ccc} -T & & \\ & -T & \\ & & 2T \end{array}\right)$$ (1)
- This contribution to the hyperfine interaction depends directly on the distance between electron and nucleus.

**Figure 1.**
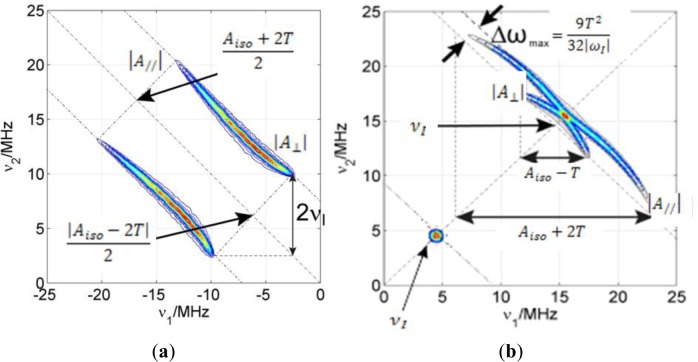
Sketch of a HYSCORE spectrum for (**a**) strong coupling case for a system with *S* =1/2 and *I* = 1/2; ν_I_ = 3.6 MHz; *T* = 7.5 MHz and *A*_iso_ = 20 MHz; (**b**) weak coupling case, ν_I_ = 14.6 MHz; *T* = 8 MHz and *A*_iso_ = 2 MHz. Additionally, the matrix peak on the diagonal corresponds to very weak couplings with nuclei whose nuclear Zeeman frequency is ν_I_ = 4.8 MHz.

If there is considerable spin density located on an atom with a magnetic nucleus, the hyperfine couplings are resolved in the CW EPR spectra as splittings of the lines (as in NMR). For example, the EPR absorption line of the electron spin in a nitroxide radical is split in three lines due to the interaction with the ^14^N nucleus (*I* = 1) in the nitroxide moiety. However, small hyperfine couplings are usually not resolved in the CW EPR spectrum. In order to measure these small couplings, pulse EPR techniques can be applied. From these, ESEEM is particularly well suited to detect small anisotropic couplings which involve mixing of the states. In particular, the 2D ESEEM experiment HYSCORE offers increased resolution particularly needed if the sample is disordered and the electron spin has couplings with many different nuclei with similar nuclear Zeeman interactions. In Figure 1, a basic notion on how to read a HYSCORE spectrum is given.

HYSCORE signals emerge in pairs symmetric with respect to the diagonal of the quadrant. Strong couplings (|*a*_iso_ + *T*| > 2|ν_I_|) appear in the left quadrant (−,+) on lines parallel to the diagonal whose separation depends on the nuclear Zeeman frequency of the nucleus. On the other hand, weak couplings (|*a*_iso_ + *T*| < 2|ν*_I_*|) appear in the right quadrant (+,+) on lines perpendicular to the diagonal (antidiagonals) that cross it very close to the nuclear Zeeman frequency of the particular nuclei. Very weakly coupled nuclei (remote) show only as peaks on the diagonal of the (+,+) quadrant exactly at their nuclear Zeeman frequency (matrix peaks). The intensity of these peaks is proportional to the inverse power of six of the distance between electron and nucleus (1/*r*^6^) and to the number of nuclei found at a certain distance. The matrix peak intensity is therefore sensitive to the spatial distribution of nuclei.

## 3. Results and Discussion

### 3.1. Addressing the Polymer-Clay Interface from the Surfactant Layer

#### 3.1.1. Incorporation of the Amphiphilic Spin Label to the Surfactant Layer

**Figure 2.**
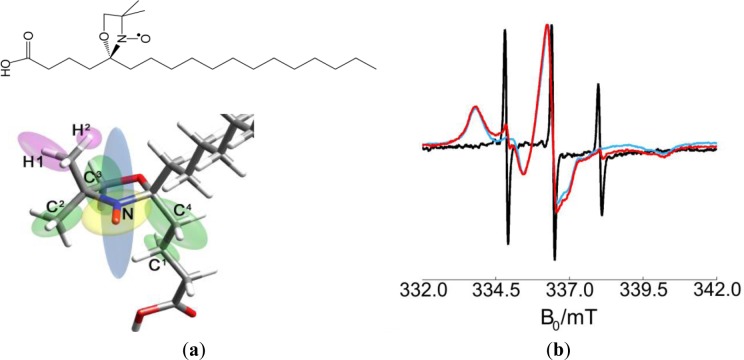
(**a**) Structure formula and stick model of 5-DSA. The numbering of the atoms corresponds to Table 2. The ellipsoids represent the directions and principal values of the nuclear spin coupling tensors; (**b**) CW EPR spectra at room temperature of 5-DSA in SDS micelles (black), NBR dispersion (blue) and NBR/Laponite RDS dispersion (red).

The free electron contained in the nitroxide group of a DOXYL ring was selected as a spin probe. Stearic acid labeled with a DOXYL ring in positions 5 or 16 (from now 5- or 16-DSA) was used to direct the spin to the surfactant layer. Due to its affinity to SDS, the spin probe was easily dispersed in the surfactant and incorporated in the polymer micelles received from the producer. Figure 2 shows the structure formula of 5-DSA. The CW EPR spectrum at room temperature of the spin probe 5-DSA in SDS micelles, and two examples for a polymer dispersion and a polymer-clay dispersion are also displayed. The spectrum in SDS micelles shows three narrow, derivative-shaped, almost equidistant lines very characteristic of a motion-averaged nitroxide, which indicates that the label undergoes very fast tumbling motion. On the other hand, when the label is added to polymer dispersions, it shows a different CW EPR spectrum with broader lines and shapes that indicate a non-mobile nitroxide, and thus a slow-tumbling motion of the probe compatible with being incorporated to a much bigger object, the polymer micelle. Addition of exfoliated clay to the dispersion did not produce significant further changes in the spectra.

#### 3.1.2. Dynamics of the Surfactant Layer Studied by Temperature Dependence of CW EPR Spectra

**Figure 3.**
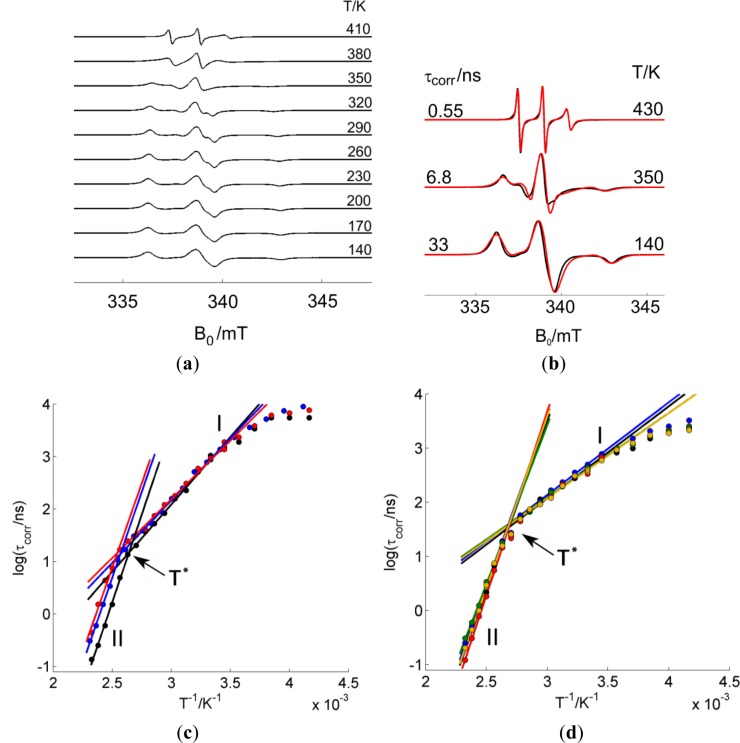
(**a**) Temperature dependence of the CW spectra of a nanocomposite prepared with PMMA-co-Bu and Laponite B; (**b**) spectra (black) at selected temperatures and their corresponding simulations (red line) obtained using the model of isotropic dynamics; (**c**) Arrhenius plot of pure NBR and nanocomposites prepared with NBR, pure rubber (black), Laponite B (blue), Laponite RD (red); (**d**) Arrhenius plot of polymer films and nanocomposites prepared with PMMA-co-Bu, pure polymer (black), Laponite B (blue), Laponite RD (red), Laponite RDS (green) and SOMASIF ME-100 (yellow). The straight lines are linear regressions to the experimental points in the high- and low-temperature regions (labeled I and II, respectively). For every composite, the intersection of the linear regressions I and II defines the transition temperature T*.

In order to obtain information about the dynamics in the surfactant layer, PCNCs were prepared with 5-DSA. The remarkable thermal stability of the spin probe allowed the acquisition of CW EPR spectra from 140 to 410 K. Below this range, there were no changes in the spectra, and above, there was decomposition of the spin label. Figure 3a shows, as an example, the thermal evolution of labeled PMMA-co-Bu/Laponite RD. A single dynamic component is found in all spectra. The ones taken at lower temperatures are close to the rigid limit. The one taken at the highest temperature—410 K, below the melting temperature of the polymer (433 K)—is very similar to the one obtained for the spin probe in dispersion at room temperature. The spectra were fitted with a home written Matlab program based on the function *chili* of EasySpin [16] assuming isotropic molecular dynamics (see Figure 3b). This is a rough model for the true dynamics, but the difference between experimentally observed spectra and spectra simulated by this simple model is not large enough to justify the use of a model with more adjustable parameters. This model also has the advantage that the time scale of the motion can be characterized by a single parameter, the rotational correlation time, τ_corr_.

Figure 3c,d shows the rotational correlation times obtained from best fit of the experiment to simulations, plotted as a function of the inverse temperature for different kinds of PCNCs. One can observe two different dynamic regimes: one at the high temperature range (low values of inverse temperature, region II), where the rotational correlation time decreases quickly with inverse temperature (note the logarithmic scale), and the low-temperature range (higher values of inverse temperature, region I) with smaller increments of τ_corr_. The correlation times obtained from spectral fits of experiments below 270 K (1/*T* > 0.0037) are not shown in the plot since they are very uncertain. At these temperatures, the spectrum is close to the rigid limit and is almost insensitive to changes in τ_corr_; therefore, we will avoid discussion of the data obtained on this temperature range. For the two temperature regions seen in the plot, the temperature dependence of τ_corr_ can be fitted to an Arrhenius law:

$$\log\tau_{\text{corr}}=\log A+\frac{E_{a}}{RT}$$ (2)

where R is the universal gas constant. The activation energy *E*_a_ can be obtained from linear fits, which are also shown. We determine the transition temperature (T*) as the intersection of the linear regressions for the two temperature ranges since it is situated in the temperature region where the transition between two dynamic regimes takes place.

Table 1 collects the activation energies *E*_a_^I^ and *E*_a_^II^ for the two regions and T* obtained from CW EPR measurements. For some of the films, the glass transition temperature (*T*_g_) was obtained by differential scanning calorimetry. The results are also collected in the table.

The temperature evolution of CW EPR spectra of surfactant spin labels were shown to be sensitive to several temperature activated processes occurring in the surfactant layer or polymeric region such as order-disorder transition of the surfactant layer, melting of the crystalline polymer domains or glass transition of the polymer [11]. Since our polymers do not have crystalline domains at any temperature and their glass transition temperature, as it was measured by DSC (Differential Scanning Calorimetry), is close to the temperature range where EPR looses sensitivity, we can rule out that these are the processes causing the observed dynamic change. Therefore, we tentatively assign the kink in the temperature dependence of τ_corr_ to the order-disorder transition of the surfactant layer, as it was already observed for melt-intercalated PCNCs [7] and organically modified clay dispersions [9].

In Table 1, we can see that for the materials produced from PMMA-co-Bu dispersions, the surfactant spin label dynamics is not changed significantly depending on whether or not there are clay nanoparticles, nor does it depend on the nature or size of the clay. On the other hand, for the composites obtained from NBR dispersions, significant changes are observed in T*; the transition temperature is higher than in the corresponding pure rubber film. The increase in transition temperature would mean that the addition of clay favors the order of the surfactant layer.

#### 3.1.3. Cationic Region Studied by HYSCORE Spectra

The HYSCORE spectra of the spin probes reveal a variety of nuclear spins coupled to the electron spin, some of which are in the nitroxide molecule and some in the close environment of the probe. In order to assign the signals to specific nuclei, 5-DSA was studied in a micellar aqueous SDS dispersion to obtain information about the probe in the absence of the polymer/clay matrix. Additionally, theoretical DFT calculations of the g-tensor as well as hyperfine and quadrupole tensors for intramolecular couplings were performed (see Table 2).

**Figure 4.**
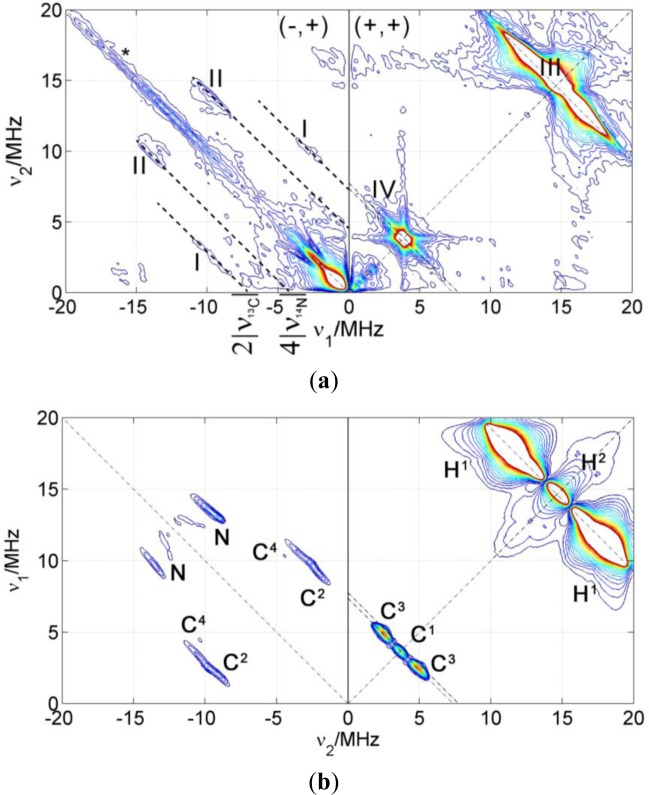

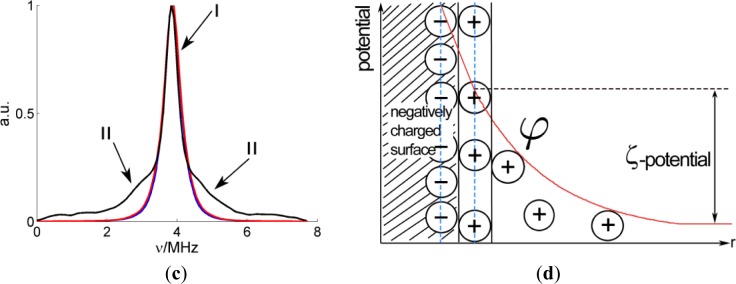
(**a**) HYSCORE spectrum of 5-DSA a water SDS micelle dispersion mixed with 50% glycerol. Experimental parameters, T = 50 K; τ = 128 ns; B = 3480 G. This magnetic field value corresponds to the center of the spectrum, hence no detectable orientation selection occurred; (**b**) simulations of the spectrum in (**a**) using the hyperfine coupling parameters listed in Table 2. (**c**) Cut of the HYSCORE spectrum depicted in (**a**) along the antidiagonal line at 3.87 MHz. The center line (I) is due to remote ^23^Na nuclei and the wings (II) are due to sodium atoms directly coordinating the nitroxide moiety. The red line corresponds to the simulation according to the Boltzmann-Poisson distribution of Na^+^ ions. (**d**) Model of the electric potential φ in the solution as a function of the distance to the negatively charged surface.

Figure 4 shows a HYSCORE spectrum of 5-DSA in an SDS micelle dispersion mixed in a 1:1 ratio with glycerol to form a good glass upon freezing. The spectrum shows signals of strongly and weakly coupled nuclei that appear in the (−,+) and in the (+,+) quadrant, respectively. The most intense feature in the spectrum is a ridge perpendicular to the diagonal in the (+,+) quadrant that crosses the diagonal at about 15 MHz (labeled as III in the spectrum), the nuclear Zeeman frequency of the protons. This signal was assigned with the help of the theoretical calculations to the protons of the methyl substituents of the DOXYL ring.

The intense peak and the long ridge along the diagonal of the (−,+) quadrant are due to experimental imperfections. The modulation depth of the electron spin echo is only a few percent of the echo intensity and the unwanted echoes could not be completely removed from the spectrum by phase cycling. The signals labeled as (I) are visible in the (−,+) quadrant where they run symmetrically along parallel lines above and below the diagonal that intercept the axes at 2ν_13C_ = 7.74 MHz. They were assigned to strongly coupled ^13^C, an isotope that is present in the sample with natural abundance of 1.07%. DFT calculations allowed refining this assignment and attributing these signals to the carbons of two of the methyl substituents in DOXYL. The signals labeled with (II) are closer to the diagonal and can be assigned to the double quantum transitions of the nitroxide ^14^N since they lay on lines parallel to the diagonal intercepting the coordinate axes at 4ν_14N_ = 4.22 MHz. The (+,+) quadrant also shows signals of weakly coupled carbons with a nuclear Zeeman frequency of ν_13C_ = 3.68 MHz. This signal is almost covered by a very close, more intense signal assigned to couplings to ^23^Na nuclei at around ν_23Na_ = 3.87 MHz in such a way that the carbon signal is only observed as a broadening of the sodium ridge below the antidiagonal.

For simulation and assignment of the HYSCORE signals, the values obtained from the DFT calculations were sufficiently accurate (see Table 2). Only the isotropic coupling hyperfine of ^14^N had to be changed from the computed value of 25.4–35.4 MHz to fit the experimental result. The good agreement between calculation and experiment for all the other couplings is remarkable. Also, the deviation of the isotropic coupling of the nitrogen is not surprising since isotropic couplings are extremely sensitive to effects such as spin polarization of inner atomic shells that are very difficult to take into account in the theoretical computation.

Besides the hyperfine couplings with magnetic nuclei within the 5-DSA molecule, which were calculated with DFT, interactions of the electron spin with ^23^Na nuclei are clearly visible in the 2D experimental spectrum, where the strong peak on the diagonal at ν_23Na_ = 3.87 MHz corresponds to distant, weakly coupled nuclei, and the extended broad wings reveal larger couplings up to about 5 MHz. The intensity of these wings with respect to the diagonal peak can be better perceived in the cut of the spectrum made along the antidiagonal line at 3.87 MHz which is shown in Figure 4c.

Sodium ions are not part of the probe but are present in its environment. In order to interpret this signal, it seems reasonable to consider that the spin probe is inserted in the micelle with the alkyl chain residing in its apolar region.

If we assume that Na^+^ ions are homogenously distributed in the solvent, outside the micelles, the spectrum can be calculated. For this computation, which is shown normalized to the maximum intensity of the matrix peak in Figure 4c, the minimum distance of sodium to the nitroxide was set to 2.4 Å, since this is the Na–O distance found for nitroxide coordinated by Na^+^ ions [17]. As can be seen in Figure 4c, the calculation (blue trace) does not reproduce the broad flanks corresponding to larger couplings.

The model of a homogeneous Na^+^ distribution is thus rejected, which is not unexpected, since the sodium cations are probably found in a higher concentration near the micelle due to electrostatic attraction to its negative surface charge. On the other hand, the cations also have a kinetic thermal energy which tends to distribute them randomly. This interplay results in a Poisson-Boltzmann distribution of cations [18,19]. The model predicts that there is a first layer of cations adsorbed at the surface of the micelle and a second diffuse layer of mobile cations. The electric potential as a function of the distance to the surface of the micelle, φ(*r*), is depicted in Figure 4d. According to this model, the local concentration of Na^+^ as a function of the distance to the micelle surface decreases exponentially:

$$N_{{\text{Na}}^{+}}(r)={\bar{N}}_{{\text{Na}}^{+}}e^{-\frac{e\varphi (r)}{kT}}$$ (3)

where the bulk concentration of sodium (2.089 × 10^−4^ Å^−3^) can be obtained from the total concentration of the cation. The electric potential can be obtained from the zeta-potential for SDS micelles, ζ = −77 mV and the Debye length, which was calculated to be κ = 6.4 Å.

$$f(r)=\zeta e^{-r/\kappa }$$ (4)

The red curve in Figure 4c shows the spectrum simulated with this model. It has a broader central peak and flanks at the sides, but not as strong and broad as the experimental spectrum. This discrepancy cannot be avoided, even if we used a more elaborate numerical model as the one suggested in [20]. If we consider the point dipole hyperfine interaction for a sodium nucleus at a distance of 2.4 Å, the hyperfine coupling constant T would be 1.5 MHz, less than the maximum observed splitting (about 5 MHz). This means that there must be an additional contribution to the hyperfine coupling, probably isotropic coupling, which is related to electron spin population in s shells of the sodium. This sort of interaction is then related to sodium ions directly coordinated to the N–O group of the nitroxide.

This finding is not easy to interpret in terms of SDS micelle structure due to the weak order governing the system SDS/5-DSA. If the polar head group of 5-DSA were aligned with the negatively charged surface of the micelle and the sodium atoms only encountered beyond it, as one would expect from a simple micelle model, the minimum distance between the nitroxide and sodium nucleus would be 6 Å. This situation is inconsistent with the experiment. Hence, at least some of the probe molecules do not have their polar head group aligned with the negatively charged surface of the micelle, but are protruding out of it. Note that the octadecyl chain of the surfactant spin probe is significantly longer than the dodecyl chains of SDS. Alternatively, sodium ions might be coordinated to the nitroxide group of the DOXYL moiety in a polar pocket inside the micelle. This appears less likely as it requires separation of Na^+^ from counterions at a distance of several angstroms across a medium with only moderate dielectric constant.

##### Dried Composites

**Figure 5.**
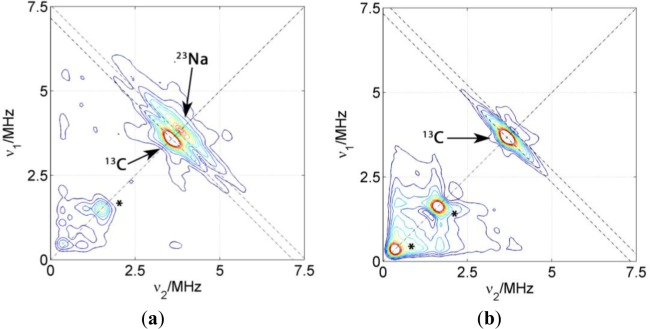
HYSCORE spectra of PCNCs prepared with 5-DSA and Laponite RD (**a**) nanocomposite prepared with NBR; (**b**) nanocomposite prepared with PMMA-co-Bu. Experimental parameters: T = 50K; τ = 128 ns; B = 3501 G. Artifacts are marked with an asterisk (*).

Figure 5 shows the HYSCORE spectra of PCNCs of different composition prepared by slow drying of the polymer/clay dispersion. Only the (+,+) quadrants are displayed since the quadrants (−,+) exclusively show couplings of the electron spin to nuclei within the DSA molecule. The dominating features of these spectra are matrix peaks lying on the diagonal line of the spectrum at the nuclear Zeeman frequencies of ^13^C, ^23^Na and, for some of the spectra, also at lower frequencies. These peaks at lower frequencies are artifacts due to either imperfect phase cycling or poor background correction. Hence, we will concentrate on the carbon and sodium region and not discuss them further. As it was already observed in the spectrum of the probe in micelles, couplings attributed to carbon 3 are detected as a ridge extending along the carbon antidiagonally. For the two kinds of PCNC prepared with Laponite RD shown in Figure 5, only the one containing NBR displayed signals reflecting considerable coupling between electron spin and ^23^Na nuclei as reflected by the extended flanks following the antidiagonal at ν_Na_. In the case of the nanocomposite prepared with PMMA-co-Bu, only a very weak matrix peak could be found for ^23^Na. Experiments with PCNC using different kinds of clay nanoparticles have revealed that the presence of ^23^Na signals does not correlate with the nature or size of the nanoparticle (the same behavior is observed even in polymer films without clay), it is exclusively related to the nature of the polymer. These results indicate that the nitroxide group of the surfactant spin probe is inserted completely in the surfactant layer in PMMA-co-Bu composites whereas it is in contact with the polar region, and in particular with sodium atoms, in NBR composites. This probably indicates a higher affinity of the alkyl chain to the PMMA-co-Bu than to the rubbers.

##### Coagulated Composites

**Figure 6.**
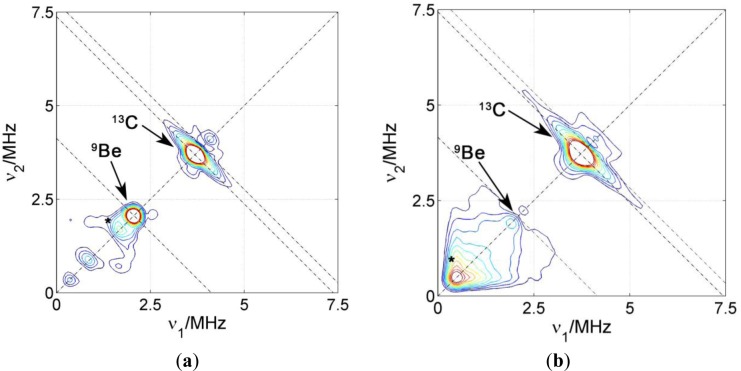
HYSCORE spectra of PCNC prepared with NBR and SOMASIF ME-100 using BeCl_2_ for coagulation. (**a**) Spin probe 5-DSA; (**b**) spin probe 16-DSA. Experimental parameters B = 3480 G; T = 50 K; τ = 128 ns. Asterisks mark artifacts in the spectra.

The surfactant interface in PCNCs prepared by coagulation of the polymer and clay dispersion upon addition of bi- or tri-valent cations was also addressed by introducing 5-DSA in the polymer micelles. For these samples, and in order to compare the environment in the surfactant layer close to the polymeric phase, another spin probe was introduced in the samples. This 16-DSA probe has the same DOXYL paramagnetic group, but in position 16, close to the end of the alkyl carbon chain. Figure 6 shows two representative HYSCORE spectra of two nanocomposites prepared with NBR, the clay Somasif ME-100 and the two spin probes located at different depths of the surfactant layer.

In industrial processes, the coagulation of rubber lattices is often induced by Ca^2+^. However, in this study, we have used Be^2+^ since ^9^Be with nuclear spin I = 3/2 and 100% natural abundance is a convenient nuclear spin probe. In both spectra, again, the matrix peak with two flanks at ν_13C_ = 3.7 MHz can be seen and assigned to carbon couplings within the probe. The two spin probes have a very similar spin density distribution and it is therefore not surprising that they have very similar ^13^C couplings. Above the carbon ridge there is a very weak ^23^Na matrix peak in the sample prepared with 5-DSA, which is almost undetectable in the one prepared with 16-DSA. On the other hand, for 5-DSA an intense matrix peak is found at ν_9Be_ = 2.1 MHz but no flanks indicating considerable coupling of the spin probe to beryllium nuclei. For 16-DSA, no remarkable ^9^Be signal was found. This shows that the coagulating Be^2+^ reside close to the surfactant head group layer, but, unlike for the Na^+^ ions in dried dispersions, we find no evidence for direct coordination to the nitroxide groups.

The decrease in the ^23^Na signals for 5-DSA when going from dried to coagulated PCNCs indicates that the sodium ions located at the surface of the SDS layer in polymer micelles are replaced by beryllium ions during coagulation. These results are consistent with the model proposing that coagulation is caused by lowering of the ζ-potential of the micelles, which is in turn due to compensation of electric charge at the surface by highly charged cations. The lack of strong hyperfine couplings for Be^2+^, as opposed to the case of Na^+^, can be understood taking into account that the stronger water coordination to Be^2+^ than to Na^+^ makes it less likely that Be^2+^ directly coordinates the nitroxide group which has a lower electric dipole moment than water. For the probe 16-DSA, the absence of any ^9^Be signal is interpreted as a consequence of the longer distance of this probe to micelle surface and to the ionic region. Additionally, this indicates that the surfactant layer keeps polymer and polar regions separated after coagulation with no considerable penetration of clay or ions into this layer.

We did not observe interaction of the 5-DSA or 16-DSA spin probes in any of the PCNCs with ^27^Si, ^7^Li or ^19^F nuclei of the clay. We thus conclude that unlike the cationic surfactants in melt intercalated composites [8], the anionic surfactants in our PCNC are not lying flatly on the clay surface.

### 3.2. Addressing the Polymer-Clay Interface from the Clay Surface

#### 3.2.1. Attachment of Cationic Spin Labels to the Clay Surface

**Figure 7.**
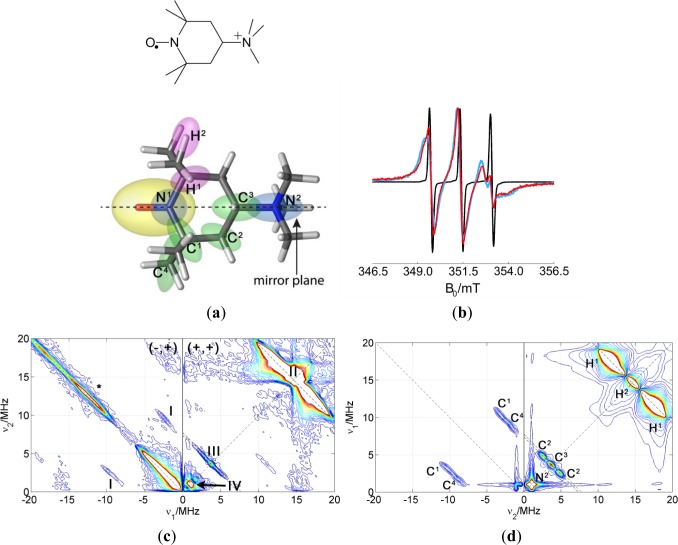
(**a**) Stick model of CAT-1. Ellipsoids represent the dipole contribution of the calculated hyperfine tensors; (**b**) CW EPR spectrum at room temperature of CAT-1 in water (black), in Laponite RDS clay dispersion (blue) and PMMA-co-Bu/Laponite RDS dispersion (red); (**c**) HYSCORE spectrum of CAT-1 in water/glycerol mixture; (**d**) simulation of the spectrum in c with the parameters collected in Table 3.

For studying the clay side of the polymer/clay interface, a nitroxide probe with a cationic group was used to address the negatively charged clay nanoparticle. The structure of this spin probe 4-trimethylammonium-2,2,6,6-tetra-methyl-piperidine-1-oxyl (CAT-1) is shown in Figure 7a. The probe has a six-membered ring with a paramagnetic nitroxide group sterically protected by two methyl groups on each C^α^-atom. The spin probe is very well soluble in polar solvents since it has a positively charged quaternary amino group. To prove the regio-specific addressing of the composites by the probe, the mobility of the spin probe was measured at room temperature in different preparations. Figure 7b shows the spectrum of CAT-1 in water (black line), whose lineshape is typical for a molecule in the fast tumbling regime. The blue line shows the spectrum of CAT-1 spin probes in a Laponite RDS dispersion. The peaks are still equidistant but they are broader, especially the one of the highest field. This spectrum corresponds to a slow down of probe dynamics due to attachment to the surface of clay nanoparticles. Note that a small fraction of the probe still exhibits the narrow line spectrum observed for the probe in water solution. This suggests equilibrium between attached and dissolved CAT-1 probe molecules. The spectrum does not significantly change upon addition of a dispersion of polymer micelles, which can be interpreted as selective attachment of the spin probe to the clay particles and not to the micellar surface. In fact, the spectrum of CAT-1 in polymer dispersion is very similar to the one in pure water (not shown).

#### 3.2.2. Study of CAT-1 Spin Probe Dispersions

Following the same strategy as for the amphiphilic probe, CAT-1 was studied in solution of water with glycerol. This provides information about the intramolecular couplings. The HYSCORE spectrum and its simulation are shown in Figure 7c,d. The most intense signals in the spectrum are the proton signals in the (+,+) quadrant and a matrix signal at ν_14N_ = 1.1 MHz. The latter signal can be assigned to the nitrogen of the quaternary amine of the spin probe since its hyperfine coupling is very small and nuclear quadrupole coupling is negligible, as expected for tetrahedral symmetry. ^14^N couplings to the nitrogen of the nitroxide group are much stronger and would only be visible (if at all) in quadrant (−,+). Again, the ridge along the diagonal of quadrant (−,+) is due to imperfect phase cycling. Some signals assigned to ^13^C appear on the antidiagonal at ν_13C_ = 3.67 MHz in quadrant (+,+) and along symmetric lines parallel to the diagonal of quadrant (−,+). Signals were again assigned to particular carbons in the molecule with the help of DFT theoretical calculations. The computed g-tensor and hyperfine and quadrupole parameters for the nuclei detected in the HYSCORE spectrum are collected in Table 3. The orientations calculated for the hyperfine tensors are represented by the orientation of the ellipsoids in Figure 7a. The anisotropic part of the hyperfine couplings is mostly axial for all nuclei and its axis points approximately to the unpaired electron, which indicated that the anisotropic part of the hyperfine tensors is dominated by the point-dipole interaction. For assignment of the signals, this contribution was crucial. In the simulations, the anisotropic hyperfine parameters were taken from the calculations and the isotropic contribution was varied until agreement with experimental data was found (value in brackets in Table 3).

**Figure 8.**
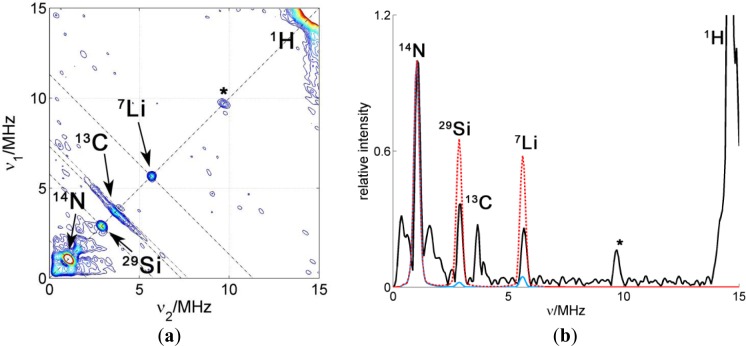
(**a**) HYSCORE spectrum of CAT-1 in a Laponite RD/water dispersion. T = 50 K, B = 3480 G, τ = 128 ns. (**b**) Diagonal cut of the spectrum in (**a**) and its simulation with different CAT-1 probe configurations. The dashed red line corresponds to a simulation with CAT-1 attached “flat” so the nitroxide lies also on the clay surface. The blue line corresponds to a simulation with only the catamine group being in contact with the surface and therefore the largest nitroxide-clay distance.

A HYSCORE spectrum of the probe CAT-1 in a clay nanoparticle dispersion is shown in Figure 8a. All the features of the probe seen in water solution are also visible in this spectrum. Additionally, two sharp matrix peaks on the diagonal are located at ν_29Si_ = 2.9 MHz and at ν_7Li_ = 5.6 MHz, which obviously arise from the interaction with nuclei in the silicate. These results demonstrate conclusively that the probes are very close to the surface of the particle, in agreement with CW EPR measurements. Remarkably, a single positive charge is sufficient to obtain this attachment of the probe to the clay particle. On the other hand, only a very weak signal was attributable to ^23^Na, although compensation of nearby clay surface charges by sodium ions would have been expected.

In order to analyze the intensity of the matrix signals, a cut of the spectrum along the diagonal was extracted to obtain the 1D spectrum shown in Figure 8b. This spectrum displays the matrix peaks. The matrix peaks arise from very weakly coupled nuclei (remote nuclei) and their intensities scale proportionally with the number of nuclei and with the sixth power of the inverse distance [15]. Taken this dependency and assuming that the number of nuclei increases roughly proportionally with the distance (since the nuclei are arranged in a plane), the intensity would decrease with the fifth inverse power of the distance. Hence, the so-called remote nuclei must not be further away than 1 or 2 nm in order to significantly contribute to the intensity of the matrix peak. To simulate the intensities of the lines in the spectrum, the crystal structure of Laponite clay reported in [21] was used taking into account that Li^+^ can substitute for Mg^2+^ in the structure. To obtain the probability for a certain position to be occupied by magnetic nuclei, ^7^Li or ^29^Si, the elementary analysis provided by the producer of the clay was used together with the natural abundance of the different nuclear isotopes. The hyperfine coupling constants were then obtained using the point-dipole approximation and the spectrum simulated using the calculated couplings. To compare simulated with experimental spectra, the simulated spectra were scaled to fit the intensity of the catamine ^14^N at ν_14N_ = 1.1 MHz. This strong intramolecular signal is conveniently used as a reference since only one nucleus per nitroxide group, with known hyperfine coupling, contributes to its intensity. The intensities of the extramolecular matrix peaks depend on the distance of the electron spin to the surface of the clay. At this point, we made calculations considering two possible models: 1. The CAT-1 molecule stands on the surface of the clay with the amino group being the only contact with the surface (blue simulation in Figure 8b) or 2. CAT-1 lies flat on the surface of the clay, with the nitroxide group also being in contact with the clay surface (red simulation). Between these two models, the distance of the electron spin to the surface differs by about 5 Å, which leads to a big difference in the peak intensities, from practically no intensity in the first case to displaying peaks almost as strong as the intramolecular ^14^N in the second case. The experimental intensities are approximately halfway in between these two limiting cases. According to this, and taking into account that the amount of unbound CAT-1 is very small according to the CW EPR, the binding geometry of the probe to the clay can be interpreted as a statistical mixture (about 50:50) of the two extremes proposed above. Note that the existence of a preferred binding geometry where the nitroxide radical is located at an intermediate distance, about 0.6 Å from the surface, would also be compatible with the experimental evidence.

#### 3.2.3. CAT-1 Spin Probe in PCNCs

The standard preparation of PCNC with CAT-1 spin probe resulted in samples with strong dipolar broadening of the nitroxide CW EPR spectrum due to spin-spin interaction of nearby probes. This interaction decreases the transverse relaxation time to a limit where no pulse EPR experiments can be performed as no echo is detected. To avoid the problem of physical proximity of the probes due to the small clay surface area, PCNC were prepared with an increased ratio of clay (17 wt% *versus* the 2–10 wt% used normally for industrial applications) which enlarges the available clay surface and thus dilutes the spin probe. These preparations still present some degree of spin-spin interaction but also a signal contribution from isolated spins on which pulse EPR experiments can be performed.

**Figure 9.**
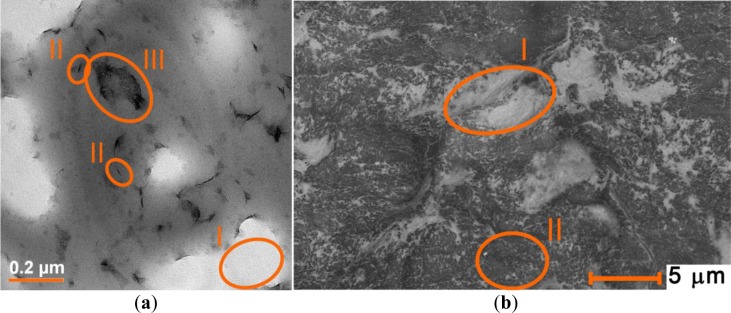
(**a**) TEM micrograph of PMMA-co-Bu/Laponite B prepared with 100 mg clay per 1 mL polymer dispersion (17 wt%). The bright areas (I) correspond to big ionic regions that have been dissolved by the procedure of sample preparation. Some exfoliated clay particles (II) and small clay agglomerations are observed (III); (**b**) SEM of the sample in (**a**). The bright areas (I) correspond to abnormally big ionic regions while dark grey areas (II) correspond to polymer regions with exfoliated clay particles.

As one can see in Figure 9, clay enriched PCNC contains abnormally large ionic regions as compared to the standard preparations. However, regions where the clay nanoparticles are isolated and the material has the desired texture are also observed. The spin-spin interactions are much more likely to occur in the first type of regions whereas the spin probes located on isolated clay nanoparticles are likely to produce a narrow signal and longer relaxation times. Therefore, pulse EPR experiments likely detect selectively those probes that are located in regions with the microstructure of interest.

##### Dried Composites

The HYSCORE spectra of nanocomposites enriched with clay show proton couplings from the molecule and the environment of the probe, as well as ^13^C- and ^14^N couplings with nuclei in the CAT-1 molecule. Besides, matrix peaks corresponding to weak couplings to ^7^Li and ^29^Si in the clay nanoparticle are found along the diagonal. Additionally, the spectra display weak and considerable couplings with ^23^Na nuclei, corresponding to remote nuclei and directly coordinated sodium atoms, respectively.

**Figure 10.**
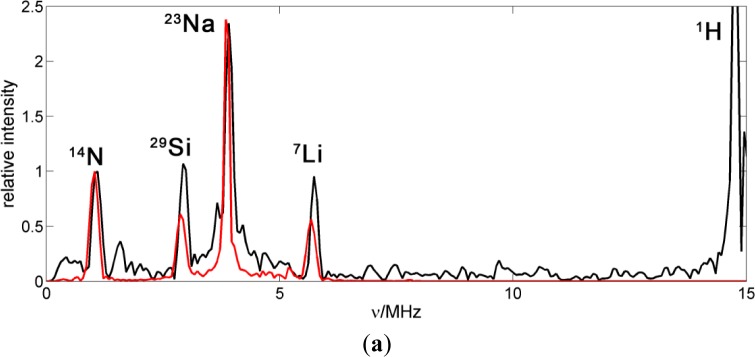

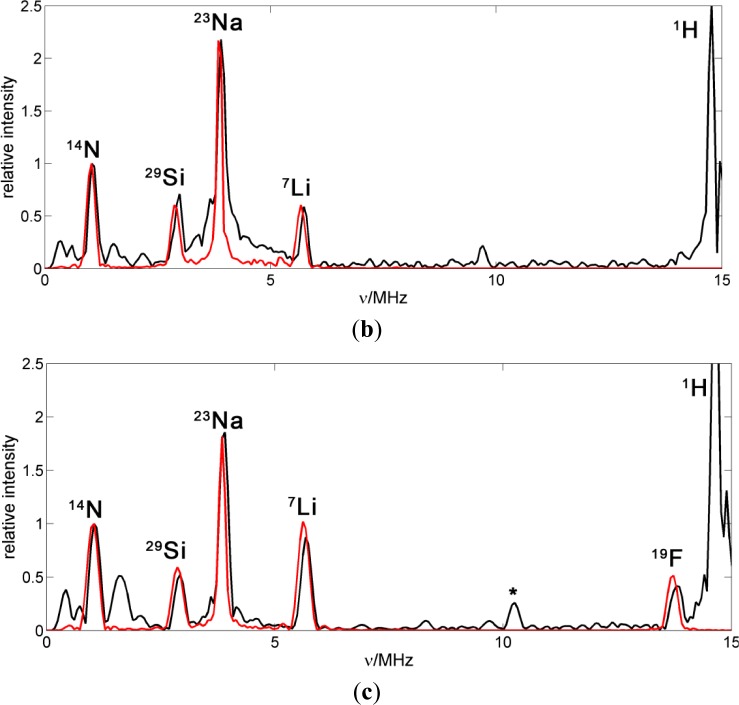
Diagonal cuts of the HYSCORE (+,+) quadrant of PCNC prepared with PMMA-co-Bu and (**a**) Laponite RD; (**b**) Laponite RDS; (**c**) Laponite B. Black traces are the experimental spectra and red traces are simulations assuming that the nitroxide moiety lies on the surface of the particle.

Again, to analyze the spectra quantitatively, the diagonal cuts were extracted and simulated assuming the CAT-1 molecule is attached to the clay surface in a way that the nitroxide electron spin is in contact with the surface. The results for PCNC prepared with PMMA-co-Bu and different kinds of Laponite are displayed in Figure 10.

All simulations were again scaled to fit the experimental intensity of the reference catamine group signal. Only the ^23^Na intensity matrix was adjusted independently in the scaling. The relative intensity of the ^29^Si and ^9^Li peaks in the simulations is very similar for nanocomposites containing RD and RDS, as expected from their very similar composition according to elementary analysis. However, the relative intensity of ^29^Si and ^9^Li peaks with respect to the ^14^N reference peak is approximately double in the sample prepared with Laponite RD than the one prepared with Laponite RDS or the simulation. This result may indicate stacking of the Laponite RD nanoparticles, where the spin probe could be sandwiched by two clay surfaces. The spectrum and simulation of the composite with Laponite RDS fit remarkably well. Note that although Laponite RDS has negatively charged phosphate groups at the narrow rims of the clay particle, no coupling with phosphorous is detected. This fact indicates that there is no preferential binding of the probe to these groups. The lines in the spectrum of the composite prepared with Laponite B have slightly different relative intensity. The ^9^Li signal is more intense, while the ^23^Na matrix peak, as well as the signal from coordinated Na, is less intense. Lower Na intensity is an unexpected finding since Laponite B has a higher cation exchange capacity (about 90–100 meq/100 g) than Laponite RD and RDS (50–60 meq/100 g). A small peak at ν_19F_ = 13.7 MHz is observed. This is due to a slightly different composition, that contains F but also a larger amount of Li.

PCNC enriched with an extra amount of clay were prepared and precipitated with a 1 M BeCl_2_ solution. The CW EPR spectra did not show line broadening but the signal was very weak, indicating that most part of the spin probe is damaged. The spectra of the probes not destroyed could be reporting from special shielded locations that do not correspond to a representative environment of clay surface and, therefore, they will not be shown or considered further.

## 4. Experimental Section

### 4.1. Preparation of Spin Probes, Polymer and Clay Dispersions and PCNCs

4-trimethylammonium-2,2,6,6-tetra-methyl-piperidine-1-oxyl (CAT-1) was purchased from Molecular Probes Inc. 5- and 16-DOXYL-stearic (5-DSA and 16-DSA) were bought from Sigma-Aldrich (St. Louis, MO, USA). 100 mM stock solutions of the spin probes were prepared by dissolving the purchased powder in water (for CAT-1) or 110 mM aqueous solution of NaOH (for 5- and 16-DSA). When not in use, the stock solutions were stored at −18 °C.

Micellar dispersions of 5-DSA were prepared by mixing 10 μL of the stock solution with 1 mL 10 wt% (0.35 M) sodium dodecyl sulfate (SDS) in a water:glycerol solution (1:1) for 16 h. The dispersion was then transferred to a quartz tube from Wilmad Labglass and frozen in liquid N_2_ where it was kept until measurement.

Laponite clay nanoparticles were kindly provided by Rockwood Additives (Cheshire, UK) and SOMASIF ME-100 by CO-OP CHEMICAL (Tokyo, Japan). Three kinds of clay nano particles were used in this work: Laponite RD, which is a pure synthetic hectorite with the sum formula NaMg_5_LiSi_8_O_20_(OH)_4_. Laponite RDS has phosphate groups attached at the perimeter of the particle as a result of a treatment with P_2_O_5_ and two fluorohectorites—Laponite B and SOMASIF ME-100—where some of the OH^−^ groups in the sum formula are substituted by F^−^ ions (sum formula Na_0.8_Mg_5.2_Li_0.8_Si_8_O_20_(OH)_4−_*_x_*F*_x_*). Table 4 reports the quantitative elemental analysis provided by Rockwood Additives for the different Laponites. The analysis is compatible within the error limit with the sum formulas. Rockwood Additives reports a cation exchange capacity (CEC) of 50–60 meq/100 g for Laponites RD and RDS and of 90–100 meq/100 g for Laponite B.

Poly-methyl-methacrylate-co-butyl-acrylate (PMMA-co-Bu) and nitrile butadiene rubber (NBR) lattices were kindly supplied by Polymer Latex GmbH (Marl, Germany) in form of turbid white dispersions. The PMMA-co-Bu latex is emulgated with SDS, whereas the NBR latex X1120 additionally contains nonionic surfactants and an antioxidant. Mean particle sizes are 123 nm for PMMA-co-Bu and 150 nm for the NBR latex. The weight ratio polymer micelles:water in the dispersions is approximately 1:1. For EPR experiments, dispersions of polymers (1 mL) as received from the producer were mixed with 10 μL of 100 mM stock solution of 5-DSA or 16-DSA. The mixture was stirred for 16 h at room temperature. To prepare polymer films without clay, the polymer dispersions were subsequently dried above the polymer glass transition temperature, at 40 °C (PMMA-co-Bu) or at 50 °C (NBR) on the surface of a Petri dish. The water evaporated slowly and the polymer film became transparent. This change in optical properties indicates interdiffusion between the different polymer particles building a continuous polymer network.

Clay dispersions were prepared by exfoliating 10 mg of clay nanoparticles in 1 mL of water by stirring for 16 h. When needed, 10 μL of CAT-1 stock solution was added to the clay dispersion, which was then mixed for 24 h.

PCNCs were prepared by mixing the polymer dispersion and the clay dispersion in a 1:1 ratio. For the drying method, films were prepared as described above for the pure lattices. The water evaporated slowly and the dispersion became a solid white at first and transparent after 2 or 3 days of drying. For studying PCNC with CAT-1, nanocomposites rich in clay were prepared by mixing 100 mg of clay with 10 μL of CAT-1 stock solution in 1 mL of water. After one day of stirring, 1 mL of micellar polymer dispersion was added, stirred further for 16 h and dried in the oven. These samples were slightly opaque; probably because of the buildup of big ionic regions with large clay agglomerates (see TEM micrograph).

PCNC samples were produced by a coagulation method adding 1 mL 1M BeCl_2_ solution to 1 mL of the polymer/clay dispersions. The composite precipitated immediately and the opaque coagulate was washed with water to remove the excess of BeCl_2_ and dried at 30 °C.

### 4.2. EPR Spectroscopy

X-band CW EPR spectra (microwave (mw) frequency of 9.5 GHz) were measured with a Bruker E500 spectrometer equipped with a variable temperature unit that was operated with liquid N_2_. The experimental conditions for the CW EPR measurements were as follows: modulation frequency 100 kHz; modulation amplitude 0.03–0.05 mT; mw power 1 mW.

All pulse EPR experiments were done at a temperature of 50 K using helium gas-flow cryostats from Oxford Instruments installed in a Bruker E580 spectrometer (Rheinstetten, Germany, mw frequency 9.8 GHz) equipped with a dielectric resonator MD5, also from Bruker.

HYSCORE experiments were performed with the standard pulse sequence π/2-τ-π/2-π-*t*_1_-π–*t*_2_–π/2-τ-echo [15]. Pulse lengths, unless stated otherwise, were 24 ns for the π/2 pulses and 16 ns for the π pulse. The value of the delay τ was typically 128 ns, as this is a blind spot for the otherwise dominating proton matrix peak. Time delays *t*_1_ and *t*_2_ were typically varied in steps of 16 ns. The whole sequence was repeated with rates between 250 and 1000 Hz, and an eight-step phase-cycle [15] was used to eliminate unwanted echoes. The value of the magnetic field was set to the centre line of the nitroxide spectrum in order to achieve maximum excitation. No orientation selection occurs under these conditions.

### 4.3. Data Processing and Spectral Simulations

HYSCORE raw data were processed by correcting the baseline of the time traces in both dimensions with a second-order polynomial function. After a Lorentz-Gaussian transformation, the traces were apodized with a Hamming function and zero-filled to a power of 2 number of-points. Finally, 2D Fourier transformation was applied to the data matrix and the absolute value of the spectrum plotted using MATLAB software (The Mathworks, Inc., Natick, MA, USA). Simulations of CW as well as HYSCORE spectra were performed using EasySpin [16].

### 4.4. Theoretical Calculations

The spin densities, EPR hyperfine and quadrupole parameters of the spin probes were calculated with the ORCA software package [22]. First, the geometry of the probe molecules was optimized by a MM2 forcefield calculation in the program ChemBio3D Ultra (Cambridge Soft, Cambridge, UK). A second geometry optimization was performed by DFT in ORCA using the B3LYP functional and a split valence basis set (SVP). The spin density distribution and EPR parameters were calculated using the B3LYP functional and EPR II the basis set [23].

### 4.5. Electron Microscopy

The electron micrographs were measured at the Electron Microscopy Center of the ETH Zurich (EMEZ). Transmission electron microscopy pictures were taken with a FEI Tecnai F30ST transmission electron microscope operating at 300 kV or with a Phillips CM12 transmission electron microscope operated at 100 kV. Scanning electron microscopy pictures were obtained using a dual beam focused ion beam scanning electron microscope (FIB/SEM) NVision from Zeiss (Oberkochen, Germany), with ESB detector.

### 4.6. Differential Scanning Calorimetry

Differential scanning calorimetry (DSC) measurements were performed in a Q200 V24.4 Build 116 from TA Instruments. The scanning range used in the experiments spanned from −90–120 °C with a scan speed of 5 °C per minute.

## 5. Conclusions

In this work, we have proved that two different regions of the polymer/clay interface can be specifically addressed with commercial nitroxide spin probes. The HYSCORE technique proved to be very useful in resolving multiple nuclei whose signals strongly overlap in a 1D ESEEM spectrum, as it has been seen with the assignment of ^13^C and ^23^Na lines, crucial for this work. According to our EPR results, the surfactant layer integrity is conserved in the PCNCs prepared by co-precipitation, in agreement with the model in which the surfactant layer keeps polymer and polar regions separated. An order-disorder transition of the surfactant is observed at around 390 K. Acrylate polymers demonstrated a better affinity for alkyl chains than the rubber, as the spin probe is aligned with the polar head of the surfactant in the acrylate nanocomposites but protrudes out of the micelle in the rubber case.

The experiments performed in PCNC precipitated with beryllium confirm the coagulation model where high-valent counterions replace the native low-valent counterions of the colloid particles. HYSCORE measurements combined with spin labeling with the catamine probe are sensitive to the clay composition and probably to stacking of clay platelets. Since no clay signals were found in the surfactant spin probe, we conclude that, unlike the alkyl chain of cationic surfactants in PCNCs prepared by melt-intercalation, the alkyl chains of co-precipitate nanocomposites are not flatly lying on the clay particle surface.

## Figures and Tables

**Table 1.** Activation energies *E*_a_^I^ and *E*_a_^II^ and transition temperatures T* obtained from CW EPR measurements. The glass transition temperatures obtained from differential scanning calorimetry *T*_g_ for the two polymers are shown for comparison.

Material	*E*^I^_a_/kJ·mol^−1^	*E*^II^_a_/kJ·mol^−1^	T*/K	*T*_g_/K
PMMA-co-Bu	14.1	51.2	372.0	283.3
PMMA-co-Bu/SOMASIF 100-ME	13.0	52.8	372.7	–
PMMA-co-Bu/Laponite RD	13.0	56.4	371.0	–
PMMA-co-Bu/Laponite RDS	12.9	48.8	371.6	–
PMMA-co-Bu/Laponite B	14.2	52.1	370.2	–
NBR	21.3	55.5	378.7	259.9
NBR/Laponite RD	18.6	53.5	393.1	260.6
NBR/Laponite B	19.7	54.4	389.5	260.0

**Table 2.** Hyperfine and quadrupole couplings obtained from the DFT calculation of the molecule 5-DSA. The orientation of the hyperfine tensors is depicted in Figure 2a. Unless another parameter is given in brackets, the same values were used to obtain good simulations of the experimental HYSCORE spectrum.

Nucleus	(*A*_x_-*a*_iso_)/MHz	(*A*_y_-*a*_iso_)/MHz	(*A*_z_-*a*_iso_)/MHz	*a*_iso_/MHz
**H^1^**	2.3	−1.3	3.8	6.2
**H^2^**	−3.4	−1.9	5.3	−1.8
**C^1^**	1.0	−0.4	−0.6	−0.5
**C^2^**	−2.0	−1.2	3.1	10.1
**C^3^**	−1.1	−0.8	1.8	2.3
**C^4^**	−2.6	−1.6	4.2	14
**N**	−25.5	−25.1	50.6	25.4 (35.4)

**Nucleus**	***Q*_x_/MHz**	***Q*_y_/MHz**	***Q*_z_/MHz**	–

**N**	0.6	1.5	−2.1	–

**Table 3.** Hyperfine and quadrupole parameters obtained from DFT calculations of CAT-1. The orientation of the hyperfine tensors is depicted in Figure 7a. These values were used to simulate the HYSCORE spectrum of the probe in water:glycerol with small adjustments in the isotropic contribution (*a*_iso_), which are shown in parenthesis.

Nucleus	(*A*_x_–*a*_iso_)/MHz	(*A*_y_–*a*_iso_)/MHz	(*A*_z_–*a*_iso_)/MHz	*a*_iso_/MHz
**H^1^**	−2.4	−1.2	3.6	5.3 (5.3)
**H^2^**	−2.9	−1.8	4.7	−1.7 (−1.7)
**C^1^**	−1.9	−1.5	3.4	−10.2 (−13.7)
**C^2^**	−0.8	−0.6	1.4	1.7 (2.1)
**C^3^**	−0.6	−0.5	1.0	−0.5 (−0.5)
**C^4^**	−1.7	−0.8	2.6	7.2 (9.2)
**N^1^**	−23.1	−22.4	45.5	32.7 (42.7)
**N^2^**	0.06	0.06	0.12	0.20 (0.20)

**Nucleus**	***Q*_x_/MHz**	***Q*_y_/MHz**	**Q_z_/MHz**	–

**N^1^**	0.7	1.4	−2.1	–
**N^2^**	0.05	0.07	−0.12	–

**Table 4.** Composition of Laponite clay nanoparticles given in wt%. Source: Rockwood Additives.

Nanoparticle	SiO_2_	MgO	Li_2_O	Na_2_O	F	P_2_O_5_	Loss	Σ
Laponite RD	59.5%	27.5%	0.8%	2.8%	–	–	8.2%	98.8%
Laponite RDS	54.5%	26.0%	0.8%	5.6%	–	4.4%	8.0%	99.3%
Laponite B	55.0%	27.0%	1.4%	3.8%	5.6%	–	7.2%	100.0%
